# cMonkey_2_: Automated, systematic, integrated detection of co-regulated gene modules for any organism

**DOI:** 10.1093/nar/gkv300

**Published:** 2015-04-14

**Authors:** David J. Reiss, Christopher L. Plaisier, Wei-Ju Wu, Nitin S. Baliga

**Affiliations:** 1 Institute for Systems Biology, 401 Terry Ave N, Seattle, WA 98109, USA; 2 Department of Microbiology, University of Washington, Seattle, WA 98103, USA

## Abstract

The cMonkey integrated biclustering algorithm identifies conditionally co-regulated modules of genes (biclusters). cMonkey integrates various orthogonal pieces of information which support evidence of gene co-regulation, and optimizes biclusters to be supported simultaneously by one or more of these prior constraints. The algorithm served as the cornerstone for constructing the first global, predictive Environmental Gene Regulatory Influence Network (EGRIN) model for a free-living cell, and has now been applied to many more organisms. However, due to its computational inefficiencies, long run-time and complexity of various input data types, cMonkey was not readily usable by the wider community. To address these primary concerns, we have significantly updated the cMonkey algorithm and refactored its implementation, improving its usability and extendibility. These improvements provide a fully functioning and user-friendly platform for building co-regulated gene modules and the tools necessary for their exploration and interpretation. We show, via three separate analyses of data for *E. coli, M. tuberculosis* and *H. sapiens*, that the updated algorithm and inclusion of novel scoring functions for new data types (e.g. ChIP-seq and transcription factor over-expression [TFOE]) improve discovery of biologically informative co-regulated modules. The complete cMonkey_2_ software package, including source code, is available at https://github.com/baliga-lab/cmonkey2.

## INTRODUCTION

It is widely acknowledged that gene regulatory networks (GRNs) are inherently modular in nature and organized hierarchically (1–3). This modular structure results from the regulation of genes by distinct combinations of regulatory factors; transcripts regulated by the same (set of) factor(s) are presumed to express similar patterns of differential expression over different cellular and environmental conditions. Such modularity is evident in GRNs across organisms, from the simplest prokaryotes to complex metazoans. Therefore, identifying co-regulated gene modules can significantly reduce the complexity of the problem of inference of genome-wide GRNs from data, and they can be exploited to greatly improve the accuracy of the inferred regulatory network topology (4–7).

For this reason, the detection of co-regulated gene modules via integrated modeling of multiple supporting data types has been an active research topic for more than a decade. Since the seminal integrated ‘module networks’ publications of Segal *et al*. (5,8), SAMBA (2) and cMonkey (9), many groups have released tools with similar overarching goals of data integration for the discovery of conditionally co-regulated modules, using various underlying statistical models and optimization methods. For example, LeMoNe (4) infers co-regulated gene modules from expression data, including detection of conditionality of co-regulation. DISTILLER (10) extends the LeMoNe framework to integrate *known* regulation with gene expression data. Like cMonkey, COALESCE (11) and Allegro (12) integrate *de novo* detection of sequence motifs with co-expression clustering to identify co-regulated gene modules and the *cis*-regulatory sequence features putatively responsible for their co-regulation. We refer the reader to a recent review (13) of integrated methods for detection of biological modules, and note the caveats presented by (14) regarding the large search-space involved (particularly for complex metazoan systems). One primary issue with all aforementioned methods and tools is the difficulty of easily extending them to new data types or organisms. Many of these are implemented as complex command-line tools or graphical user-interfaces that can only be applied to a limited pre-defined set of model organisms (typically, a few metazoans [human, mouse, fruit fly, etc.], and often *E. coli* and *S. cerevisiae*).

The cMonkey integrated biclustering algorithm (9) was designed to decipher, from genome-wide measurements, the conditional co-regulation of genes by integrating different types of information which support evidence for their co-regulation and effectively constrain, or regularize, the complex search space mentioned above. In the end, cMonkey produces biclusters that are constrained by one or more of these streams of data (9), over subsets of experimental measurements. The three primary data sources that were originally integrated and optimized by cMonkey were (i) transcript co-expression (similarity of expression profiles) of clustered genes across subsets of measurements; (ii) *de novo* detection of common putative *cis*-acting gene regulatory motifs (which we will hereafter abbreviate as GREs, for gene regulatory elements) in the promoters of clustered genes (putative binding locations of the same transcriptional regulators); and (iii) significant connectivity between clustered genes in functional association or physical interaction networks (implying meaningful functional association, which is often correlated with co-regulation). We used cMonkey to construct a Environmental Gene Regulatory Influence Network (EGRIN) model (15) for *Halobacterium salinarum NRC-1*, and have more recently used it to construct EGRIN models for many more organisms covering all three branches in the tree of life ((7,16–19), and unpublished). Work on the cMonkey algorithm and its implementation has been ongoing during that time, and we are now releasing a completely updated and reengineered version of the cMonkey software tool, with optimized performance, improved documentation and ease-of-use for end-users, and enhanced modularity which will make it straightforward to extend by interested developers.

The primary algorithmic modification in the new implementation is that it uses a global optimization, rather than the local, individual cluster optimization utilized by the original procedure. Additional algorithm updates include changes to the individual scoring scheme for subnetwork clustering, as well as to the heuristic used to integrate the different scores. All of these changes, which serve to improve the procedure's runtime performance by roughly 3-fold, result in additional benefits which we will elucidate below. We have reimplemented the updated algorithm into a new framework, called cMonkey_2_, which improves ease-of-use for the end-user; greatly simplifies automated integration of additional data types and scoring mechanisms; and enhances the resulting output to facilitate visualization and exploration of biclusters and their associated evidence (e.g. *de novo* predicted GREs) in the context of other databases and web services. These improvements make cMonkey_2_ a fully functioning, unified platform for integrating many kinds of genome-wide data to build gene co-regulatory modules, plus the necessary tools to explore and use them to inform biological insights.

## MATERIALS AND METHODS

Hereon, we refer to the originally published version of cMonkey as cMonkey_1_. For a detailed overview of the cMonkey_1_ algorithm and its data integration model, we refer the reader to (9).

### cMonkey_2_ algorithm modifications

In the following we describe only the relevant and notable algorithm changes which have been made in the updated version, which we call cMonkey_2_. These include (i) a switch from local bicluster optimization to a global optimization procedure; (ii) a switch from a probabilistic association network-based score to a network density-based scoring function; (iii) a modified, more efficient heuristic for combining the three model components into an integrated clustering score while enabling stochastic exploration of the search space. Although all three of these modifications appear to replace rigorous statistical models and distributions with heuristics, as we will show, the practical effect is a significant decrease in algorithm run-time with no detriment to performance (to the contrary, cMonkey_2_ actually achieves improvement in performance).

cMonkey_1_ is a local optimization procedure, in which biclusters are *seeded*, one at a time, and then optimized individually. As each additional bicluster is generated and optimized, any overlap (in the form of gene membership) between it and previously-optimized biclusters was reduced by constraining the number of biclusters (default expected value $v$ = 2) into which each gene may fall. The most significant modification we have made to the algorithm is that cMonkey_2_ instead performs a global optimization, that is modeled on the simple, widely-used and effective *k*-means clustering algorithm (20). After beginning with a chosen distance metric and an initial partitioning of all genes into exactly *k* clusters ($v$ = 1 cluster per gene), the basic *k*-means algorithm iterates between two steps until convergence: (i) (re-)assign each gene to the cluster with the closest centroid and (ii) update the centroids of each modified cluster. The updated cMonkey_2_ algorithm performs an analogous set of moves with four primary distinctions relative to *k*-means: (i) the distance of each gene to the centroid of each cluster is computed using a measure that combines condition-specific expression profile similarity, similarity of putative GREs detected in gene promoters, and connectedness in one or more gene association networks (and/or additional scoring measures added via the new modular plug-in framework; see below); (ii) each gene can be (re-)assigned to more than one cluster (default $v$ = 2); (iii) at each step, conditions (in addition to genes) are moved among biclusters to improve their cohesiveness; and (iv) at each step, genes and conditions are not always assigned to the most appropriate clusters. We now elaborate upon these four details.

In cMonkey_2_, as with standard *k*-means (as well as the original cMonkey_1_), *k* must be chosen *a priori*; by default, cMonkey_2_ sets *k* such that each bicluster will contain ∼20 genes on average (thus, given that each gene is assigned to $v$ = 2 biclusters by default, *k* is set to *N_g_* × $v$/20, where *N_g_* is the number of transcripts measured across all experiments). cMonkey_2_ begins each iteration with a set of bicluster memberships *m_i_* for each element (gene or condition) *i*, where by default |*m_i_*| = $v$ = 2 for genes (as described previously), and |*m_i_*| = *N_c_*/2 for conditions (*N_c_* is the number of conditions, or measurements, in the expression data set; note that for standard *k*-means clustering, |*m_i_*| = 1 for genes and |*m_i_*| = *N_c_* for conditions). cMonkey_2_ then computes log-likelihood score matrices *R_ij_*, *S_ij_* and *T_ij_*, for membership of each element *i* in each bicluster *j* based upon, respectively, co-expression with the current gene members (**R**), similarity of GREs in gene promoters (**S**), and connectivity of genes in networks (**T**). For the network scores (**T**), the original procedure computed a *p*-value for enrichment of network edges among genes in each bicluster using the cumulative hypergeometric distribution. This computation was inefficient, and moreover could not account for weighted edges in the input networks, so we replaced it in cMonkey_2_ with a more standard weighted network clustering coefficient (20) evaluated only over the genes within each bicluster.

Following computation of the individual component scores, cMonkey_2_ computes a score matrix *M_ij_* that contains the integrated score (a weighted sum of log-likelihoods, as in cMonkey_1_) supporting the inclusion of gene *i* in bicluster *j*. At this stage cMonkey_1_ would then train an ‘iteratively-reweighted constrained logistic regression’ on each bicluster's *M*_.*j*_ to obtain a posterior probability distribution *p_ij_*, to classify potential bicluster members *i* based upon these scores. This procedure proved to be a significant bottleneck on algorithm performance. In cMonkey_2_ we instead compute a kernel-based cumulative density distribution from these scores, to estimate the relative probability *p_ij_* that each element *i* belongs in each cluster *j*. The width of the density distribution kernel is set dynamically to be larger for smaller (fewer gene) biclusters, so as to increase the tendency to add genes to small biclusters, rather than remove them. Whereas cMonkey_1_ would then sample elements *i* from *p_ij_* to stochastically add or remove elements from each bicluster *j*, in the new implementation cMonkey_2_ we instead add a small amount of normally-distributed random ‘noise’ to the scores *M_ij_* in order to achieve a similar type of stochasticity (which helps prevent the algorithm from falling into local minima; this noise decreases during the run to zero at the final iteration). The result of this noise is that at the beginning of a cMonkey_2_ run, biclusters are rather poorly defined (co-expression, for example, is poor), but during the course of a full set of 2000 iterations, as this noise is decreased, the biclusters settle into a much more significant set of minima (Supplementary Figure S1).

At the end of each iteration, cMonkey_2_ chooses a random subset of genes or conditions *i*, and moves *i* into bicluster *j* if, for any biclusters *j*′ which it is already a member, $p_{ij} >p_{ij^{\prime }}, \forall j^{\prime }$, and out of the corresponding worse bicluster *j*′ for which $p_{ij} >p_{ij^{\prime }}$. Thus, as with the *k*-means clustering algorithm, cMonkey_2_ performs a global optimization of all biclusters by moving elements among biclusters to improve each element's membership scores, rather than by optimizing each bicluster one-at-a-time (as cMonkey_1_ did). Note, however, that we have introduced an added degree of stochasticity to the optimization procedure, both from the selection of a random subset of genes and conditions to be moved at each iteration, and from the randomization described above. This type of metaheuristic does not exist in the standard *k*-means clustering algorithm.

### The cMonkey_2_ tool: implementation details

We have completely re-implemented the cMonkey_2_ software tool, transforming it into a data integration platform that enables non-technical researchers to easily analyze their gene expression data in the context of additional evidence, while allowing developers to extend and tailor the base functionality with minimal development effort. cMonkey_2_ is available as a command-line-driven Python application. All aspects of the implementation center on modularity and extensibility, enabling developers to easily incorporate their novel data types and/or scoring methodologies into the procedure. For data input and integration, the tool now downloads, automatically from external databases, relevant information for nearly any microbe, including genome data and gene annotations (currently using NCBI parsed annotations from RSAT (21); Microbes Online (22)); gene functional associations (STRING (23)), expression data (Gene Expression Omnibus (GEO) (24)) and others (e.g. DoE KBase).

We implemented cMonkey_2_ in object-oriented Python, in order to more effectively modularize and streamline the codebase. This switch alleviated all of the major speed and memory bottlenecks which were causing difficulty with the original implementation in GNU R.

**Figure 1.**
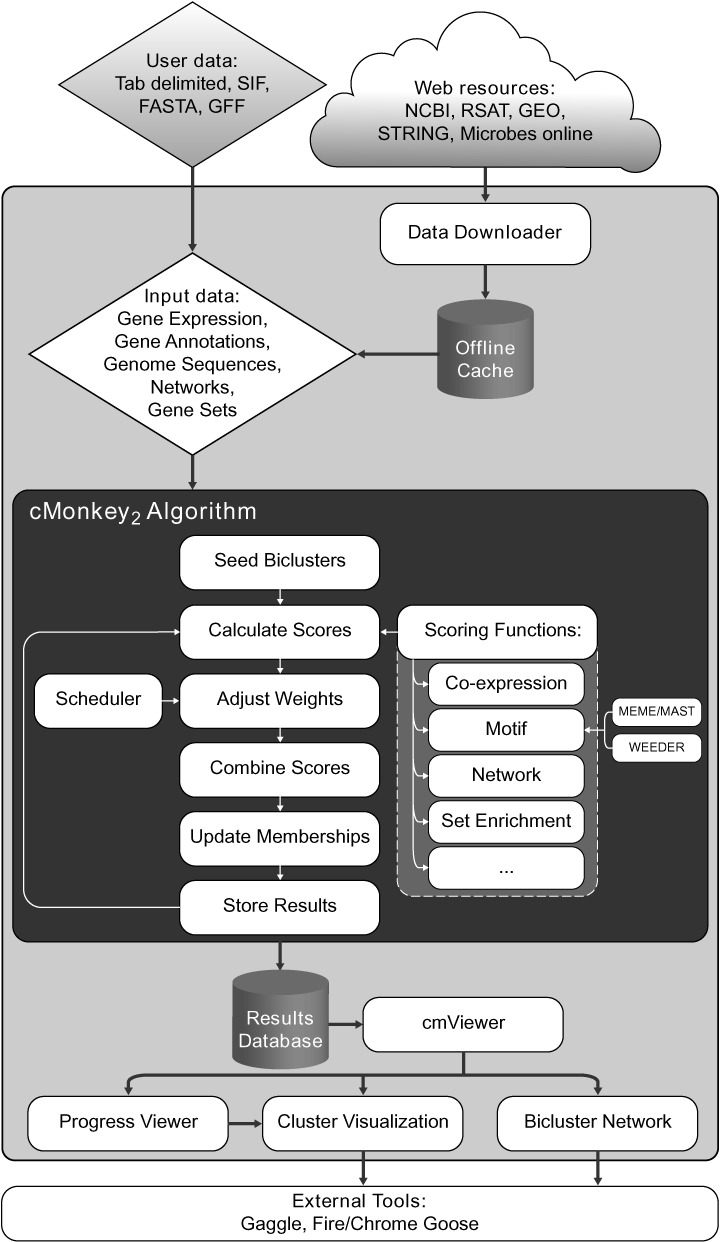
The cMonkey_2_ data integration and analysis pipeline. Only expression data (*User data*) is required as input by the user. All other input data, if not provided by the user, is fetched automatically from online services (*Web resources*). All of these data are passed into the cMonkey_2_ algorithm (dark gray area) which seeds, and then iteratively refines biclusters by integrating the output of various modularized scoring functions, following a weighting schedule (*Scheduler*) which may be customized by the user. Resulting clusters are stored in a database which may be queried by the *cmViewer* tool to view progress and visualize results.

For a typical cMonkey_2_ run, the user provides a file containing a set of mRNA expression log-ratios in standard tab-delimited text file format. The user will also typically provide a three-letter KEGG organism code (e.g. ‘eco’ for *E. coli*), which is used to identify and automatically fetch additional information (genome sequence, annotations, operon predictions, promoter sequences, functional associations) from various scientific databases (21–23). For sequenced prokaryotes and unicellular eukaryotes (such as *S. cerevisiae*), cMonkey_2_ provides solutions to common tasks such as name mapping and abstraction of organism-specific aspects including genomic information. Upon initialization, the tool downloads (if necessary) and caches the relevant public data locally. For example, predicted operons (25) are downloaded from Microbes Online (22), full genome sequences and gene annotations are fetched from NCBI via RSAT (21), and these data are used to parse out predicted promoter sequences for each annotated transcript (Figure 1). In cases where the organism of interest is not yet included in these databases (e.g. it is newly sequenced), custom versions of these files may be supplied in standard formats (e.g. tab-delimited, GFF, FASTA, etc.) to enable motif searching and network clustering. If these additional data are not available, biclustering on the expression data *only* is still possible, and will be performed by default.

#### cMonkey_2_ scoring functions

At the core of a cMonkey_2_ computation lies a cMonkey run. A run consists of a set of input data and configuration parameters and a set of scoring algorithms that are activated at certain iterations of the optimization procedure (i.e. following a schedule), and combined using user-specified weights. All parameters have defaults which have been configured to work well with all test cases, data sets and organisms tested (at this point, over twenty). The user can override certain configuration parameters or methods to customize the run. The configuration parameters may be set via command-line or a hierarchy of user-defined and default configuration files.

At the heart of the cMonkey run is a scheduler that executes specific scoring functions at user-defined iterations and with a user-defined scoring weight. Each scoring function computes the corresponding *k* × |*i*| scores for associating a given gene/condition *i* with each of the *k* biclusters. An important detail is that these scores are not required to be comprised of standard distance measures which may be difficult or expensive to calculate. They can alternatively simply consist of a (relative) measure of prior expectation that gene/condition *i* belong in cluster *k* given the data and given the other genes/conditions in the cluster. An example of such an *ad hoc* scoring mechanism is delineated in detail in our description of the new set-enrichment scoring function (see below).

After computation of all scoring matrices, cMonkey_2_ integrates the scores, as described in more detail below, via a combiner function, which runs a list of scoring functions in sequence and combines their results according to specified weights. Bicluster memberships (rows and columns) are then updated based upon these combined scores. A cMonkey_2_ scoring function has a standard interface, so users can implement and run an arbitrary number of differently-weighted scoring functions based upon user preferences. The user has the choice to override the parameters of the scoring functions, via command-line options and/or configuration files. Default scoring functions for the three standard cMonkey data types—co-expression, MEME-detected conserved promoter GREs and network clustering—are provided by default. Below, we describe implementations and use-cases for two additional, newly implemented scoring functions.

### Implementation and integration of novel row scoring functions

To demonstrate the utility and ease of integrating additional streams of evidence for gene co-regulation via implementation of new scoring mechanism (as described above), we implemented two additional row (gene) scoring functions which were not part of the original cMonkey_1_ algorithm. The first of these integrates a ‘set-enrichment’ scoring function, which enables the user to influence bicluster optimization to enrich for user-defined sets of genes (e.g. similar gene functional annotations; known promoter binding mapped via ChIP or RNase hypersensitivity; or known GREs). The second scoring function integrates an additional motif detection algorithm (Weeder (26)) to add motif detection via enriched *k*-mers, and to search for motifs in regions other than gene promoters (here, we use 3’ UTRs)—a functionality complementary to that already provided by MEME. Below, we describe the motivation and implementation of these two scoring functions. Later, in the Results section, we present an analysis of their influence on cMonkey_2_ biclustering results on data sets for three different organisms.

#### Set-enrichment row scoring function

The set enrichment scoring function was developed to easily incorporate (and enrich biclusters for) predefined gene sets. Given a file which lists annotations or groupings of genes into (possibly overlapping) sets with unique identifiers, the set enrichment scoring function computes, at each iteration, the significance of overlap between each bicluster's member genes and the genes annotated for each set using the Fisher's exact test. For each bicluster, the set with the most significant overlap (smallest *p*-value) is chosen for training, and a set of row scores is generated that increases the probability of retaining cluster genes or adding new genes that are the enriched set. The gene scores are computed by a simple heuristic in which we multiply the log_10_ of the aforementioned *p*-value by 1.0 for genes which are in the bicluster *and* are members of the enriched set; by 0.5 for genes which are in the set but *are not* in the bicluster; and by 0.0 for all other genes. To increase stability of the set enrichment approach we added a low-pass filter which sets the *p*-value to a minimum of the Bonferroni cutoff *p*-value given the number of sets tested. These final gene (row) scores are then normalized and combined with the other scoring functions to train cMonkey_2_ biclusters.

#### Weeder motif detection row scoring function for detection of enriched *k*-mers

While MEME (27) has been shown (28,29) to be among the most sensitive and robust sequence motif detection algorithms available, the fact that it models GREs as position-specific scoring matrices (PSSMs) and uses expectation maximization to detect the most overrepresented signatures in a set of input sequences means that it is more sensitive to detecting certain types of motifs. The Weeder algorithm (26) searches for overrepresented degenerate *k*-mers (rather than PSSMs), and has been shown (28) to be quite sensitive to detecting other types of regulatory motif signatures, including miRNA binding sites in mammalian genomes (30). Thus, in order to detect putative signatures for miRNAs in human genomes that might be associated with disease, we developed a cMonkey_2_ scoring function that integrates Weeder motif detection and optimization into the algorithm. This custom row scoring function replaces MEME with Weeder to search for enriched motifs in gene promoters (using default parameters, in ‘medium’ search mode on both strands), and returns gene-specific scores assessing the significance of each promoter's match to the detected motifs. Specifically, the function records the *n* ≤ 4 highest-scoring motifs, ranked by score and provided as PSSMs by Weeder. As with the default MEME scoring function, it then provides those PSSMs as input to MAST (31) to compute the significance (sequence *p*-values) of the match of each gene's promoter to the detected motif(s). These sequence *p*-values are then used by the remainder of the cMonkey_2_ pipeline identically to the default MEME calculation.

### cMonkey_2_ output, monitoring and visualization and exploration

Provenance is a key concern in the new implementation, and all information necessary to completely reproduce a given run is stored in the output, including code version, input parameters and configuration files and random number seeds. cMonkey_2_ provides a web interface to the output cMonkey_2_ results database, served via an embedded web server, that enables a user to observe the progress of the run, via histograms of scores and optimization progress of mean statistics at each iteration (Supplementary Figure S4(A)). The design of this monitoring implementation as an embedded web server allows cMonkey_2_ analyses to be run on a remote server (e.g. Amazon's EC2 platform) and monitored using a local web browser. In the future, we intend to extend this interface to enable initialization and control over cMonkey_2_ runs, as well as remote storage, visualization and exploration of computation results.

The interface also enables searching, selection and viewing of individual biclusters during a run, and upon completion, a Cytoscape (32) network visualization for exploring relationships between genes, detected GREs, and their membership in biclusters (Supplementary Figure S4(B)). All of these resources are populated with FireGoose (33)/ChromeGoose XML microformats, which enable easy integration and exploration using other external tools and databases via the Gaggle (34), such as MeV (35) for expression analysis, STAMP (36) and RegPrecise/RegPredict (37,38) for motif comparisons, DAVID (39) for function analysis, KEGG (40) for pathway analysis, among many others. Additionally, we have implemented an interface which enables recorded exploration of biclusters via an interactive IPython notebook.

### Methods for evaluation of cMonkey_2_ module detection

#### *E. coli*: Comparisons to other published module detection algorithms

In order to assess the ramifications of the algorithm changes which we made to cMonkey_2_, we evaluated its performance relative to both cMonkey_1_, to other popular clustering methods—*k*-means (41) and WGCNA (42), and to published data integration/module detection algorithms COALESCE (11), DISTILLER (10) and LeMoNe (4). We note that this list is by no means comprehensive, but is rather meant to provide a representative sampling of the various data integration and module detection algorithms available. For all algorithms, we used an *E. coli* gene expression compendium, containing 868 measurements of mRNA expression for 4203 genes, compiled and normalized by (10). To quantify algorithm performance, we used intrinsic (tightness of cluster co-expression; motif significance) and extrinsic (recapitulation of known biology) measures. For the intrinsic measures, we used the *mean square residue* (43) and MEME (27) motif *E*-value to quantify bicluster and detected GRE quality, respectively. For the extrinsic quality assessment, we used the RegulonDB (44) database as the gold standard for comparing with known *E. coli* regulatory network modularity and known regulation. We ran all algorithms listed, other than DISTILLER, for which we used the author-provided clusterings (based upon the same *E. coli* data set). For all algorithm runs, we attempted to generate clusters with a similar average number of genes to that of the cMonkey runs (either, for example, by adjusting *k* for *k*-means, or trying different size *k*-mers for COALESCE).

##### Comparisons of intrinsic measures of cluster quality

For the intrinsic measures of cluster and motif detection, we used cluster *mean squared residue* (43)) to quantify cluster cohesiveness across included conditions. For motif detection, we quantified the likelihood of detecting clusters with genes that contain a statistically significant putative GRE (MEME (27) *E*-value ≤ 1).

##### Detection of known *E. coli* regulons

The primary goal of cMonkey is to reconstruct, from expression data, a comprehensive set of co-regulated gene modules. We have chosen to define a co-regulated gene module as a set of genes which are regulated by the same combination of transcription factors (TFs). We assessed cMonkey and the other algorithms in their capability to recapitulate experimentally annotated *E. coli* regulons in RegulonDB (44) using precision and recall. For precision, we computed the fraction of computed biclusters that had significant gene membership overlap (more than two genes, computed via cumulative hypergeometric *p*-value, controlled for false discovery rate FDR ≤ 0.01) with at least one of the 257 such combinatorial regulons in RegulonDB. For recall, we computed the fraction of all 257 combinatorial regulons which were rediscovered by the algorithm.

A standard measure of algorithm performance relative to an incomplete gold standard is an area under the precision-recall (AUPR) curve. However given our evaluations which use a single clustering for each algorithm, we use the geometric mean of precision and recall, or *G*-measure (45). Thus, for a given number of true positives (TP), false positives (FP) and false negatives (FN),

(1) \begin{equation*} P = \frac{{\rm TP}}{{\rm TP+FP}}; R = \frac{{\rm TP}}{{\rm TP+FN}}; G = \sqrt{R\times P}. \end{equation*}

For completeness, we also report the *F*_1_ score, which is the harmonic mean of precision and recall.

##### Detection of known *E. coli* transcription factor binding sites

cMonkey has the distinction among the algorithms tested (along with COALESCE) that it infers, *de novo*, conserved putative GREs in the promoters of genes in each cluster using MEME (27) (by default), and optimizes the clusters to improve those GREs. To evaluate this aspect of cMonkey, we assessed the performance of all algorithms in identifying groups of genes containing significant combinations of *bona fide* GREs in their promoters. For each cluster generated by each algorithm, we applied MEME for motif detection *post facto*, with the same set of parameters utilized by cMonkey, to the promoters of each cluster's member genes. Using FIMO (46), we then scanned the GREs detected by MEME across the entire *E. coli* genome to identify significant (FDR ≤ 0.05) motif instances. We then compared the locations of these motif instances with 2283 experimentally-determined binding locations for 101 transcription factors (TFs) with at least three binding sites in the RegulonDB *BindingSiteSet* table. If positions of a GRE aligned to the positions of a TF significantly more often than expected at random (FDR ≤ 0.01), then we classified that GRE as a match to the TF.

As previously, we assessed each algorithm's precision (fraction of all clusters with a match to a RegulonDB TF) and recall (fraction of all 101 RegulonDB TFs independently detected), and combined these into a single *G* measure.

##### Evaluations of new set-enrichment scoring module for *E. coli*

To evaluate the efficacy of the set-enrichment module (see Methods section for details), we parsed the RegulonDB regulons into gene sets and integrated these into cMonkey_2_ using the set enrichment row scoring function. Thus, we added an additional constraint to cMonkey_2_ which allowed the algorithm to optimize biclusters that (simultaneously with the other aforementioned constraints—coexpression, GRE detection, and network connectivity) should be more consistent with the annotated *E. coli* regulons.

#### *Mycobacterium tuberculosis*: Integration of ChIP-seq and TF overexpression via set-enrichment

The newly-introduced cMonkey_2_ set-enrichment scoring function was developed to improve the capability of cMonkey_2_ to construct co-regulated gene modules which are simultaneously enriched for known gene sets. By enriching clusters for gene sets which are expected to include additional evidence for co-regulation (e.g. regulons from ChIP-chip/seq or known regulons; functional annotation such as Gene Ontology; or co-regulation via some other pre-computed evidence type). To further test the capability of the cMonkey_2_ set-enrichment scoring function to improve detection of experimentally validated regulons, we investigated its influence on modules detected for *Mycobacterium tuberculosis*, using a large gene expression compendium and new global ChIP-seq and transcription factor overexpression (TFOE) measurements.

##### *M. tuberculosis* data

We used a compendium of 2,325 publicly available *Mycobacterium tuberculosis* transcriptome measurements collated from TBDB (http://tbdb.org), as described in (18). For our set-enrichment assessment, we integrated genome-wide binding measurements for 154 *M. tuberculosis* TFs, assayed via ChIP-seq (47), and transcriptome measurements following induction (over-expression; hereafter, TFOE) of 206 TFs (48). As described in (18), from the ChIP-seq measurements we distilled 7,248 significant TF-gene interactions through significant binding in regions spanning -150 to +70 nucleotides around transcriptional start sites for 142 TFs. Similarly, for the TFOE measurements, we used RNA measurements from the same cultures in which the TFs were induced for ChIP-Seq to obtain transcriptomes resulting from overexpression of 205 TFs. From these measurements, we identified 3,785 mRNAs with significant expression change (*p*-value ≤ 0.01).

##### Running cMonkey_2_ on *M. tuberculosis*

We ran cMonkey_2_ on the *M*. tuberculosis data in eight different combinations: (i) without the ChIP-seq or TFOE gene sets (default); (ii) with only the ChIP-seq gene sets via the set-enrichment scoring function; (iii) with only the TFOE gene sets via the set-enrichment scoring function; and (iv) both ChIP-seq and TFOE gene sets, weighted equally via the standard cMonkey_2_ weighting mechanism. We additionally ran cMonkey_2_ with motif detection/integration turned off for all four combinations. For all runs, we used *k* = 600, as in (18), but excluded the (default) inclusion of EMBL STRING functional co-association networks, in order to eliminate the possibility of redundancy between test- and training data. Given the non-deterministic optimization of cMonkey_2_, we ran each combination of parameterizations ten times, enabling us to report estimates of the mean and standard errors to facilitate comparisons. In total, this investigation comprised 80 separate cMonkey_2_ runs.

##### Recovery of gene sets significantly enriched in cMonkey_2_ modules

For each run, we used a Benjamini-Hochberg-corrected *p*-value (*p*-value ≤ 0.01) to identify the total number of biclusters (out of 600) which were significantly enriched for any of the 142 ChIP-seq gene sets or any of the 205 TFOE gene sets.

#### Human Lung Squamous Cell Carcinoma (LUSC)

To test the capability of the new cMonkey_2_ set-enrichment and Weeder scoring functions to improve detection of validated regulons in mammalian systems, we investigated their performance on The Cancer Genome Atlas (TCGA) lung squamous cell carcinoma data set. In particular, we assessed the recovery of miRNA regulators (using 3’ UTR sequences as input for training to the Weeder scoring function, and a pre-computed database of miRNA to target gene predictions as training input for the set-enrichment function).

##### Human LUSC data

We downloaded RNA-seq gene level counts for 20 351 genes across 475 lung squamous cell carcinoma (LUSC) tumors (49) from the October 17, 2014 run of the Broad TCGA GDAC Firehose (doi:10.7908/C1CJ8CFD). Using DESeq2 (50) we normalized the RNA-seq gene level counts using the variance-stabilizing transformation, computed the coefficient of variation for each gene, and selected the top 2000 genes with largest coefficients of variation as input for cMonkey_2_. Each cMonkey_2_ run detected 133 biclusters. Significant co-expression for each bicluster was ensured by filtering out all biclusters where the variance explained by the first principal component was less than the variance explained by the first principle component for randomly sampled gene sets of same size for more than 5% of the random samples. This filtering led to an average of 112 ± 8 biclusters per cMonkey_2_ run on the LUSC tumors.

##### Running cMonkey_2_ on LUSC tumors

We used GeneMANIA (51,52) as the gene-gene interaction network training input for human cMonkey_2_ runs. A gene synonym thesaurus was created from the Ensembl BioMart database to covert between different gene identifiers. Weeder was used to discover motifs in the 3’ UTR sequences that were extracted from the UCSC genome browser FTP site using the same methods we have described previously (30,53). The TargetScan database of predicted human miRNA target genes release 6.2 (54) was used for set-enrichment training. We ran cMonkey_2_ on the LUSC normalized RNA-seq gene expression using three different training approaches: (i) no cis-regulatory training inputs (i.e. no *de novo* motif detection); (ii) training on *de novo*-detected 3’ UTR Weeder motifs; and (iii) training only on set-enrichment using TargetScan miRNA target gene predictions. As cMonkey_2_ is non-deterministic in nature we ran each of the three training approaches six times to provide estimates of the mean and standard errors to facilitate comparisons.

##### Human LUSC: comparing number of significant weeder motifs

The significance of Weeder motifs was determined by calculating empirical *p*-values for each bicluster Weeder motif score using pre-computed Weeder motifs scores from 1000 randomly sampled gene sets. As bicluster sizes vary we precomputed gene sets sizes from 5 to 65 genes on the interval of 5 genes and selected the closest gene set size for empirical *p*-value calculation. A Weeder motif score was considered to be significant if it had an empirical *p*-value less than or equal to 0.05. A post-hoc Weeder motif discovery and empirical *p*-value calculation was conducted to determine the number of significant motifs detected for cMonkey_2_ runs that did not train on Weeder motifs. A Student's *t*-test was used to compare the number of significant Weeder motifs between the three approaches.

##### Recovery of miRNAs implicated in LUSC

Each bicluster was tested for enrichment of miRNA target genes from the TargetScan database of predicted human miRNA target genes as described previously (53). For each run we determined the overlap of the miRNAs enriched in biclusters with the manually curated miR2Disease database which implicates 110 different miRNAs in the etiology of lung cancer and/or non-small cell lung cancer. A Student's *t*-test was used to compare the number of LUSC miRNAs re-discovered between the three approaches.

## RESULTS

Below, we summarize results of our three separate analyses described in the Methods section. Overall, cMonkey_2_ proves to be a worthy successor to cMonkey_1_, and, based upon our assessments of both cluster quality and recapitulation of known modularity and mechanisms in prokaryotic gene regulatory networks, is an excellent tool for this purpose. We also would like to note that these comparisons may be supplemented by our previously-published evaluations of cMonkey_1_, which included extensive performance comparisons with many other biclustering methods, and an analysis of performance on randomized and shuffled data sets.

### Evaluation and comparison of module detection for *E. coli*

In the following sections, we evaluate the performance of cMonkey_2_ in recapitulating known regulation as annotated in the RegulonDB database (see Methods section for details). Results of all comparisons are summarized in Table 1 and plotted in Supplementary Figure S2.

**Table 1.** Summary of intrinsic and extrinsic measures of module construction performance on *E*. coli data set, for several algorithms, as described in **Methods** and also shown in Figure S2

							GRE			Regulon			Combi-Regulon^(7)^		
Method	*k*^(1)^	<*E* ≤ 1 >^(2)^	<*N_g_* >^(3)^	<resid >^(4)^	$\left<\frac{N_{\rm{cl}}}{\rm{Gene}}\right>$^(5)^	Gene Cvg.^(6)^	Recall	Prec.	*G*	Recall	Prec.	*G*	Recall	Prec.	*G*
**cMonkey_2_**	400	0.69	21.0	0.61	2.0	1.00	0.61	0.29	0.39	0.89	0.52	0.65	0.79	0.51	0.62
**no motif**	400	0.13	21.0	0.60	2.0	1.00	0.43	0.21	0.28	0.80	0.46	0.58	0.72	0.46	0.56
**no net**	400	0.75	21.0	0.61	2.0	1.00	0.63	0.31	0.42	0.82	0.47	0.60	0.73	0.49	0.59
**no mot, net**	400	0.11	21.0	0.60	2.0	1.00	0.43	0.21	0.28	0.60	0.30	0.40	0.49	0.28	0.35
**set-enrich**	400	0.71	21.0	0.61	2.0	1.00	0.60	0.28	0.38	0.94	0.55	0.69	0.79	0.59	0.68
**cMonkey_1_**	400	0.96	25.7	0.68	2.6	0.94	0.52	0.24	0.33	0.64	0.42	0.51	0.50	0.42	0.46
**DISTILLER**	150	0.09	19.1	0.64	1.2	0.57	0.38	0.43	0.40	0.41	0.55	0.47	0.27	0.31	0.28
**LeMoNe^(8)^**	64	0.34	60.0	0.70	1.0	0.92	0.17	0.34	0.23	0.26	0.34	0.29	0.23	0.38	0.28
**LeMoNe^(8)^**	373	0.27	60.5	0.63	6.9	0.78	0.42	0.23	0.30	0.66	0.53	0.59	0.62	0.54	0.58
**COALESCE^(9)^**	175	0.38	99.5	0.64	5.1	0.81	0.24	0.25	0.24	0.60	0.58	0.59	0.50	0.61	0.55
**WGCNA**	25	0.52	168.0	0.73	1.0	1.00	0.11	0.48	0.18	0.09	0.28	0.13	0.11	0.36	0.17
***k*-means**	213	0.12	19.7	0.66	1.0	1.00	0.44	0.32	0.37	0.51	0.32	0.39	0.43	0.32	0.37

*Notes*: ^(1)^ Total number of clusters/modules detected. ^(2)^ Fraction of clusters containing a detected motif with MEME *E*-value ≤ 1. ^(3)^ Mean number of genes over all clusters. ^(4)^ Mean cluster mean squared residue. ^(5)^ Mean number of clusters per gene. ^(6)^ Gene coverage: fraction of all genes included in at least one cluster. ^(7)^ ‘Combi-Regulon’ is short for combinatorial regulon (see **Methods**). ^(8)^ Default LeMoNe parameters except minimum cluster size of 3. ^(9)^ COALESCE *k*-mer length of 7 (default) or 8.

#### Intrinsic measures of cluster quality

When compared to the algorithms tested (see Methods section, we found that cMonkey_2_ identified clusters with, on average, tighter co-expression (cluster *mean squared residue* (43)), and, other than cMonkey_1_, with a greater likelihood of containing a statistically significant GRE (MEME (27) *E*-value ≤ 1) (Table 1 and Supplementary Figure S2). Typically, there is, somewhat paradoxically, a tradeoff between obtaining tight co-expression and detecting significant GREs. Thus it is noteworthy that cMonkey_2_ obtained tighter clusters, while still detecting more clusters with more statistically significant GREs. While cMonkey_1_ clusters were more likely to contain a significant motif (96%), this is primarily because it is both (a) training more heavily on GREs than on expression data, which explains the less coherent (higher residual) cMonkey_1_ biclusters; and (b) redundantly detecting the same significant GREs in multiple redundant clusters (i.e. achieving greater precision at the expense of lower recall). This also explains the reason that even cMonkey_2_ (no motif), for example, achieved greater recall (and hence greater *G* score) than cMonkey_1_. The modified algorithm of cMonkey_2_, which only allows each gene to be assigned to no more than two biclusters, is far more stringent than the probabilistic constraint in cMonkey_1_. A similar effect explains the greater precision of some other algorithms (e.g. WGCNA) than cMonkey—the discovery of relatively fewer (e.g. only 25 by WGCNA) and significantly larger (∼8 × larger, for WGCNA) modules, enables it to focus on only the most significant (and thus most easily characterized) modules.

#### Detection of known *E. coli* regulons

Surprisingly, cMonkey_2_ detected combinatorial regulons with substantially greater precision (51% vs. 42%) *and* recall (79% vs. 50%) than cMonkey_1_. In fact, cMonkey_2_ achieved greater recall than all algorithms tested, and greater precision than all except COALESCE (54%). cMonkey_2_ achieved the greatest *G* score (see Methods section) for combinatorial regulon detection vs. RegulonDB (Table 1 and Supplementary Figure S2; using the *F*_1_ score instead (Methods section) produces the same outcome). If we instead compare performance in recovering standard regulons (as opposed to combinatorial regulons; see Methods section), cMonkey_2_ again achieved the greatest *G* (and *F*_1_). It is noteworthy that cMonkey_2_ surpassed even DISTILLER on these combined measures, even though DISTILLER uses known regulon data as part of its training set, which gives it greater precision.

#### Detection of known *E. coli* GREs

cMonkey_2_ again outperformed cMonkey_1_ in both precision and recall for detection of validated GRE sites in RegulonDB (Table 1 and Figure S2; see Methods section). It also achieved greater recall than all other assessed methods, although with relatively lower precision than COALESCE. However, cMonkey_2_ surpassed all methods in the combined precision-recall *G* (and *F*_1_) measure. While DISTILLER achieved greater performance than the original cMonkey_1_ in these measures on our *E. coli* GRE detection benchmarks, primarily due to its greater precision, our analysis reveals that the algorithm modifications in cMonkey_2_ have enabled it to outperform all methods.

#### Evaluation of cMonkey_2_ integration of motifs and networks for *E. coli*

In order to evaluate whether the data integration scheme of cMonkey_2_ performed as expected, we included results for runs of cMonkey_2_ in which motifs and/or networks were not included as part of the training data. Not surprisingly, using the full complement of data performs significantly better than excluding motif information. However, we found that excluding only the network data (here, STRING (23) functional association links) did not significantly handicap the algorithm; although excluding both networks *and* motifs performed significantly worse than only excluding either data type separately.

#### Enrichment for *E. coli* known regulons via new set-enrichment row scoring module

We will now present the results of integrating the two novel cMonkey_2_ scoring modules (described in Methods section) in more detail and evaluate their utility in improving the method's performance in recapitulating RegulonDB regulons and known GREs.

Table 1 and Figure S2 shows that this integration effectively improved the cMonkey_2_ recapitulation of RegulonDB regulons and combinatorial regulons (particularly, the precision of regulon detection), while not significantly impeding its ability to meet the other default constraints of tight conditional co-expression and significant GRE detection. Clearly, the degree to which this module can improve these measures (and by result decrease other measures) depends upon adjustment of its weighting schedule. While we acknowledge the circularity of this assessment, it proves that the set enrichment row scoring module has the intended effect and could be effectively used to integrate ChIP, functional annotations, or related data types into the cMonkey_2_ module detection process.

### Evaluation of co-regulated modules detected for *Mycobacterium tuberculosis*

All assessments of cMonkey_2_ module predictions for *M. tuberculosis* are summarized in Table 2 and Supplementary Figure S3.

**Table 2.** Summary of cMonkey_2_ module construction on *M*. tuberculosis expression data set, including varying combinations of additional prior data (ChIP-seq and TFOE; see **Methods**) via the set-enrichment scoring function

	Expression		ChIP-Seq^(*a*)^		TFOE^(*a*)^		Both^(*a*)^	
	Motif	No Motif	Motif	No Motif	Motif	No Motif	Motif	No Motif
**mean residual**	0.53 ± 0.01	0.49 ± 0.01	0.53 ± 0.01	0.49 ± 0.01	0.53 ± 0.01	0.50 ± 0.01	0.53 ± 0.01	0.50 ± 0.01
**mean motif log**_10_ p**-val**.	-9.14 ± 0.07	–	-9.21 ± 0.10	–	-9.19 ± 0.08	–	-9.31 ± 0.12	–
**clusters w. motif***E* ≤ 1	564.8 ± 7.6	–	565.3 ± 7.2	–	563.0 ± 2.9	–	571.5 ± 3.4	–
**ChIP-seq**								
**clusters signif**.^(1)^	349.5 ± 5.8	187.3 ± 7.7	397.5 ± 8.5	418.1 ± 30.4	349.9 ± 11.2	182.8 ± 9.9	395.3 ± 8.9	419.2 ± 11.9
**TFs signif**.^(2)^	142.0 ± 0.0	133.4 ± 3.5	142.0 ± 0.0	141.5 ± 1.0	142.0 ± 0.0	129.9 ± 4.8	142.0 ± 0.0	142.0 ± 0.0
**unique clusters signif**.^(1)^	95.7 ± 3.2	87.1 ± 3.9	97.6 ± 4.3	116.2 ± 2.5	93.0 ± 3.8	88.1 ± 4.0	98.3 ± 3.9	118.4 ± 4.3
**unique TFs signif**.^(2)^	135.6 ± 1.8	134.7 ± 2.9	137.0 ± 1.3	136.5 ± 1.5	136.3 ± 1.6	136.2 ± 1.2	136.5 ± 1.7	135.8 ± 3.3
**TFOE**								
**clusters signif**.^(1)^	346.3 ± 7.8	249.1 ± 9.7	347.8 ± 12.5	255.2 ± 10.3	402.2 ± 11.3	485.5 ± 15.8	413.1 ± 14.9	491.1 ± 7.2
**TFs signif**.^(3)^	198.8 ± 1.8	179.0 ± 3.9	200.0 ± 1.9	181.4 ± 5.6	201.6 ± 1.7	199.5 ± 1.8	202.8 ± 1.0	199.8 ± 5.4
**unique clusters signif**.^(1)^	138.9 ± 3.8	136.2 ± 5.3	135.6 ± 2.4	137.2 ± 5.7	144.4 ± 3.5	174.9 ± 4.6	146.3 ± 8.2	171.9 ± 4.2
**unique TFs signif**.^(3)^	191.7 ± 2.7	189.2 ± 3.4	191.6 ± 2.6	188.4 ± 3.2	189.6 ± 2.9	190.8 ± 2.3	191.0 ± 5.0	190.7 ± 2.5

Shown are statistics regarding recapitulation of TF target gene sets in the ChIP-seq and TFOE measurements, for cMonkey_2_ runs on data with inclusion of varying prior information. The values in the table rows labeled ‘clusters signif.’ (number of significant clusters) correspond to those in the bar chart of Figure S3. The rows labeled ‘Unique clusters/TFs signif.’ denote clusters/TFs with a single unique match to a TF/cluster, respectively, and respectively represent precision and recall.

*Notes*: ^(*a*)^ All runs included expression data as well. ^(1)^ Out of a total of 600 clusters predicted. ^(2)^ Out of 142 TFs tested via ChIP-seq. ^(3)^ Out of 205 TFs tested via overexpression (TFOE).

#### Set-enrichment scoring function significantly increases recovery of set-enriched modules

Recently we reported the construction of a global gene regulatory network for *Mycobacterium tuberculosis* (Mtb) by applying cMonkey to 2,325 publicly available Mtb transcriptome profiles (18). We previously validated this predicted network using two separate global data sets: (i) genome-wide binding locations for 143 TFs measured via ChIP-seq (47), and (ii) global transcriptional consequences of overexpressing 206 TFs (TFOE) (48). We hypothesized that training on these experimentally determined TF-regulated genes from ChIP-seq or TFOE would improve cMonkey_2_ gene regulatory module inference. We tested this hypothesis by applying cMonkey_2_ analysis to the same transcriptome profiles for Mtb as used previously (18), and varying the training inputs (MEME *de novo cis*-regulatory motif detection; ChIP-seq set-enrichment; and TFOE set-enrichment).

Importantly, we first found that integration of MEME motifs in cMonkey_2_ optimization significantly increased the number of modules that were enriched for both ChIP-seq (350 vs. 187 significantly enriched modules, Student's *t*-test *p*-value = 5.0 × 10^−12^, Table 2) and TFOE (346 vs. 249 significantly enriched modules, *p*-value = 6.2 × 10^−10^, Table 2) TF targets, in comparison runs in which motifs were excluded from training (i.e. co-expression alone). This result demonstrates clearly that the cMonkey_2_ integration of sequence information from MEME *de novo* motif detection significantly improves discovery of biclusters that are enriched for Mtb TFs.

Using the set-enrichment scoring function to train on ChIP-seq or TFOE (while excluding motif detection), also significantly increased the number of enriched modules beyond what was discovered by co-expression training alone (ChIP-seq *p*-value = 2.3 × 10^−9^, TFOE *p*-value = 2.5 × 10^−13^, Table 2), or to the runs trained on MEME motifs (ChIP-seq *p*-value =1.3 × 10^−7^; TFOE *p*-value = 5.8 × 10^−7^, Table 2). Notably, this improvement was achieved with relatively little decrease in bicluster co-expression (i.e. little increase in residual, Table 2), suggesting that, by integrating this alternate form of prior *cis*-regulatory information, cMonkey_2_ is effectively exploiting the complex, multifactorial biclustering search space to result in modules with similar co-expression ‘quality,’ but which are significantly more enriched with the desired sets.

Due to the indirect nature of TFOE responses (vs. inherently direct interactions measured via ChIP), the condition sensitivity of the experiments, and the noise inherent to ChIP-seq measurements, there is a small amount of overlapping cis-regulatory information between the ChIP-seq and TFOE data sets (18), which leads to little to no increase in the number of TFOE set-enriched modules detected when trained on ChIP-seq sets, and vice versa. The complementary nature of these data sets and the lack of a gold-standard set of cis-regulatory predictions meant that we did not have an independent validation that could be used to assess the strength of these predictions. We address this lack of external validation in the following section on lung squamous cell carcinoma where an external validation set is available.

### Evaluation of module detection for human Lung Squamous Cell Carcinoma (LUSC)

#### Increased recovery of LUSC-implicated miRNAs by training on Weeder motifs

Previously, we developed methods to discover miRNA mediated regulation from gene co-expression clustering by discovering 3’ UTR motifs using Weeder *post-facto* on the 3’ UTR sequences of genes in the clusters (53). The integration of Weeder into cMonkey_2_ allows us to train biclusters based simultaneously on co-expression and Weeder 3’ UTR motifs thereby increasing the potential for discovering meaningful miRNA co-regulation. We tested this hypothesis by applying cMonkey_2_ to The Cancer Genome Atlas (TCGA) lung squamous cell carcinoma (LUSC) patient tumors to discover miRNA mediated co-regulation. We observed significant increase in the number of significant 3’ UTR motifs discovered in when cMonkey_2_ is trained on Weeder motifs when compared with runs not trained on any cis-regulatory inputs (*p*-value =1.5 × 10^−3^; Table 3). Training on Weeder 3’ UTR motifs also led to a significant 2.5 fold increase in recovery of miRNAs implicated in lung cancer as compared to runs not trained on any cis-regulatory inputs (*p*-value = 6.0 × 10^−5^; Table 3). We have demonstrated that integration of training on Weeder 3’ UTR motifs into cMonkey_2_ has improved the discovery of 3’ UTR motifs, which in turn leads to an impressive increase in the discovery of disease implicated miRNAs.

**Table 3.** Summary of cMonkey_2_ module construction performance on *H*. sapiens lung squamous cell carcinoma data set, for several different training schemes, including novel Weeder-based 3’ UTR motif detection, and pre-computed miRNA target gene predictions via novel set-enrichment scoring function. See **Methods** for details.

Training Method	Number of Significant Motifs	Comparison *p*-value^(1)^	miR2Disease miRNAs	Comparison *p*-value^*a*^
**Expression only**	9.3 ± 2.1	-	3.5 ± 0.84	-
**Weeder motif training**	16 ± 3.1	1.5 × 10^−3^	8.8 ± 1.5	6.0 × 10^−5^
**Set-enrichment training**	9.5 ± 2.3	9.0 × 10^−2^	8.0 ± 0.63	1.8 × 10^−6^

*Notes*: ^(1)^ All *p*-value comparisons are relative to ‘Expression only.’

#### Increased recovery of LUSC-implicated miRNAs by set-enrichment training

A faster alternative for training cMonkey_2_ runs to discover miRNA mediated regulation in mammalian species with large genomes is set-enrichment with databases of pre-computed miRNA target gene predictions such as TargetScan (54). cMonkey_2_ was run on the TCGA LUSC patient tumors (see above) and trained, using set-enrichment, on TargetScan miRNA target gene predictions. This analysis led to a significant 2.3-fold increase in recovery of miRNAs implicated in lung cancer as compared to runs not trained on any cis-regulatory inputs (*p*-value = 1.8 × 10^−6^; Table 3). Importantly, there was not a significant difference in the number of miRNAs recovered between Weeder 3’ UTR motifs or TargetScan miRNA target gene training approaches (*p*-value = 0.24); however omitting the *de novo* motif detection resulted in a ∼3 × improvement in cMonkey_2_ run time in the TargetScan set-enrichment runs (4.7 ± 0.08) hours, versus the Weeder 3’ UTR motif runs 14.3 ± 0.2 hours; *p*-value = 9.1 × 10^−5^). These results demonstrate that if regulatory factor to target gene databases exist that set-enrichment approaches can be used in place of de novo motif detection and also lead to significant performance improvements.

## DISCUSSION

We have described cMonkey_2_, an updated and improved framework for detecting co-regulated modules of genes via automated data integration and optimization. We have described our recent modifications to the algorithm, which served to improve both its runtime performance, as well as its ability to discover optimized and experimentally validated gene regulatory modules. Based upon our tests on *E. coli*, cMonkey_2_ proves to be a strong performer in this crowded arena of regulatory network module detection and data integration.

We have completely overhauled the cMonkey_2_ implementation, focusing on ease-of-use for the end user (with automatic downloading and integration of many different data sources for any sequenced and annotated microbe), and on simplicity for the developer in integrating new data types and scoring schemes into the procedure. We demonstrated the utility of two new scoring mechanisms with use-cases for three different organisms—*E*. coli, *M*. tuberculosis and *H*. sapiens, and showed that a simple integration of a new set-enrichment scoring procedure, as well as a new motif detection algorithm (Weeder) improved upon the existing capability of cMonkey_2_ to detect valid co-regulated gene modules and cis-regulatory motifs. These tests moreover demonstrated the importance of motif integration as part of cMonkey, revealing that this constraint can significantly improve module detection performance when additional data sets (other than expression data) are not available—as would be the case, for instance, for newly-sequenced and culturable microbes with no developed genetics capability.

Because various motif detection algorithms use different statistical models and heuristics for learning them, they have different, often complementary capabilities for detecting different types of real signatures (55). For this reason, a number of researchers (56,57) have taken to integrating the predictions of several different motif detection algorithms and have demonstrated resulting increased sensitivity and/or precision on prokaryotic genomes (56,58). Our long-term goal is to integrate a number of motif detection algorithms into an ensemble learning procedure for learning co-regulated modules with cMonkey_2_. This will include searching, in addition to annotated gene promoters (the current default), their 5’ and 3’ UTRs for potential miRNA and post-transcriptional regulatory motifs. As we have shown, cMonkey_2_ now provides a straightforward framework for this type of integration, and we have identified excellent candidates (29) including BoBro (59), Spacer (60) or dyad-analysis (61), and BioProspector (62), which model DNA motifs in different ways, as additional targets for integration.

In the future, in addition to integration of multiple motif detection algorithms, we intend to use this new framework to add additional constraints via phylogenetic genomic conservation (e.g. (63) and related), as well as other new data types including genomic location constraints provided by ChIP-seq (64), ATAC-seq (65) or DNase-seq (66) measurements, which will provide significant additional constraints on the bicluster (and in particular, motif) optimization. Moreover, the framework provides the opportunity to investigate other measures of gene expression pattern similarity (e.g. mutual information) to identify other potential patterns of co-regulation.

Our desire is to see cMonkey_2_ become a focal point for a community of users and developers, with additional data types and scoring function modules being contributed by the community. To this end, development of the framework is openly hosted on Github (http://github.com/baliga-lab/cmonkey2), with extensive documentation, wikis, and discussion groups. We will moreover provide a framework for automatically testing modifications and improvements contributed by the community via benchmarks similar to the RegulonDB ones presented here.

## SUPPLEMENTARY DATA

Supplementary Data are available at NAR Online.

SUPPLEMENTARY DATA
