# Supplementary material for: cMonkey2: Automated, systematic, integrated detection of co-regulated gene modules for any organism
Source: Nucleic Acids Res. 2015 Apr 14;43(13):e87. doi: 10.1093/nar/gkv300 (PMC4513845; doi:10.1093/nar/gkv300)
Supplement: SUPPLEMENTARY DATA [file supp_43_13_e87__index.html]

cMonkey2: Automated, systematic, integrated detection of co-regulated gene modules for any organism — cMonkey2: Automated, systematic, integrated detection of co-regulated gene modules for any organism — SUPPLEMENTARY DATA 

# cMonkey2: Automated, systematic, integrated detection of co-regulated gene modules for any organism

## SUPPLEMENTARY DATA

**Files in this Data Supplement:**

- SUPPLEMENTARY DATA
