# Supplementary material for: cMonkey2: Automated, systematic, integrated detection of co-regulated gene modules for any organism
Source: Nucleic Acids Res. 2015 Apr 14;43(13):e87. doi: 10.1093/nar/gkv300 (PMC4513845; doi:10.1093/nar/gkv300)
Supplement: SUPPLEMENTARY DATA [file supp_gkv300_nar-03045-met-z-2014-File002.pdf]

SUPPLEMENTARY MATERIALS

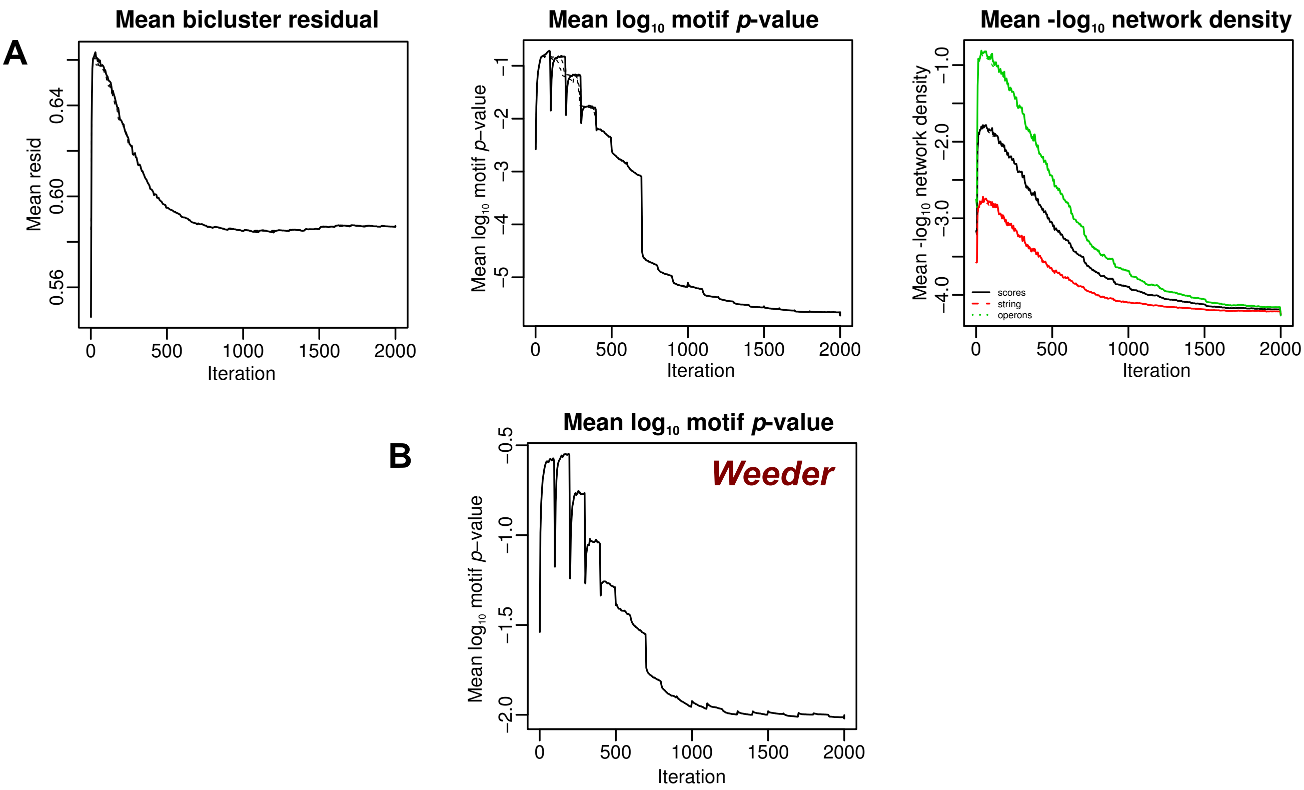

Figure S1. Simultaneous optimization of multiple co-regulation scores during a cMonkey$_2$ run. **A.** Plots of mean bicluster residual (mean square residue), mean $\log_{10}$ motif match p-value, and mean -$\log_{10}$ network density as a function of optimization iteration, during the course of a single cMonkey$_2$ run (means are taken over all biclusters during the run). In all plots, lower scores are better. **B.** Plot of mean $\log_{10}$ motif match p-value for a run in which Weeder was used for motif detection rather than MEME. The fluctuations in motif scores during early iterations is because (a) motif searching is performed only every 100 iterations, and during these early iterations, there is (b) a relatively high amount of stochasticity, and (c) a low contribution of motifs to the total bicluster score.

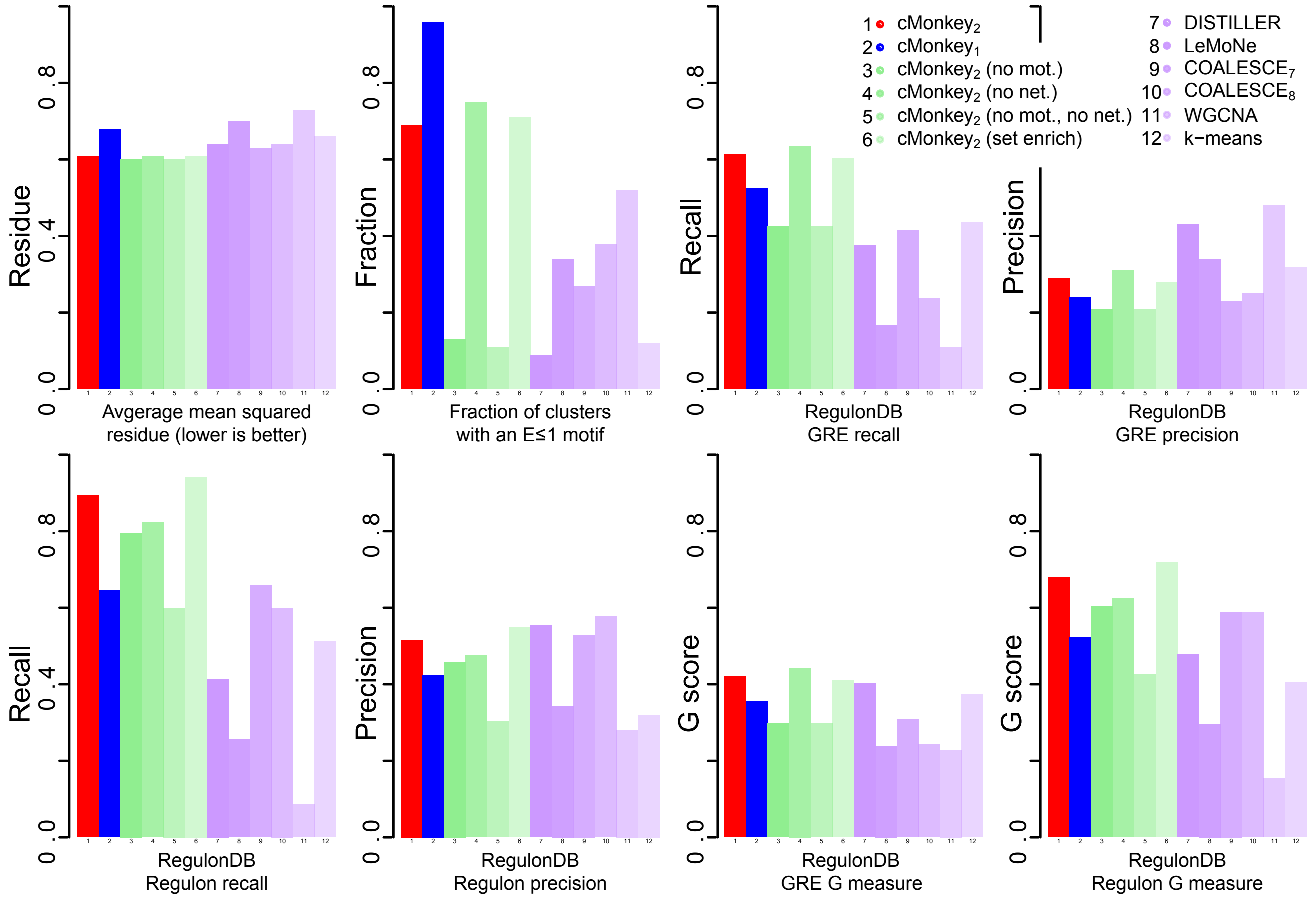

Figure S2. Evaluation and comparison of co-regulatory modules detected for *E. coli* using various intrinsic and extrinsic measures. Except where noted, for all plots, higher is better. Bar plots comparing, from top-left to bottom-right, respectively: Average cluster mean squared residue (a measure of cluster coherence; lower is better); Fraction of detected clusters with a significant MEME motif (MEME E-value ≤ 1); GRE recall and precision relative to RegulonDB; Annotated regulon recall and precision relative to RegulonDB; GRE and regulon precision/recall statistics integrated using their geometric mean (G-masure (1)).

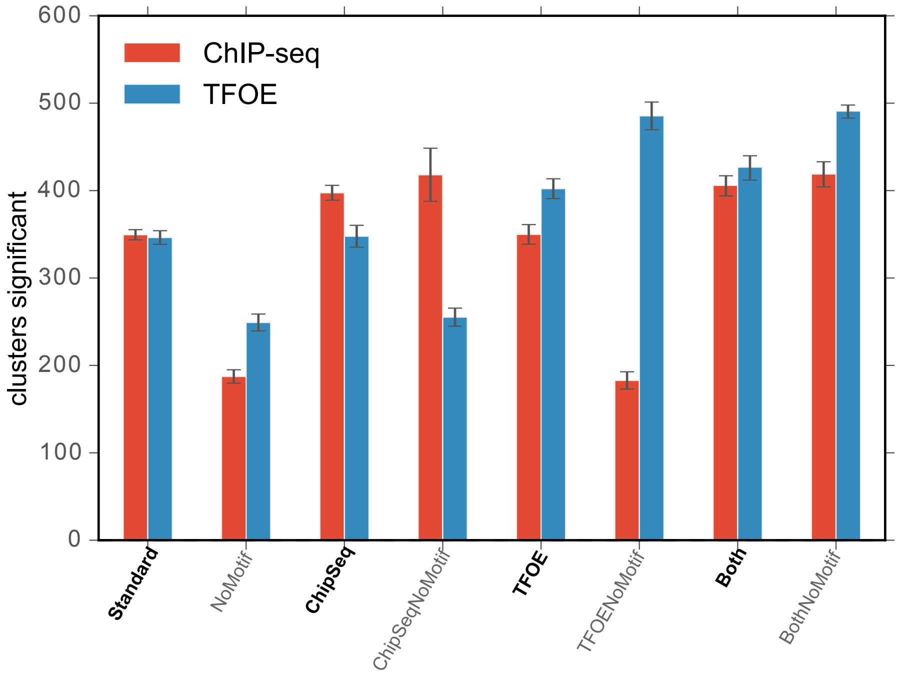

Figure S3. Evaluation and comparison of co-regulated modules detected for M. tuberculosis, including set-enrichment for ChIP-seq and TF overexpression sets. For all plots, higher is better. Total number of clusters (out of 600) significantly enriched ($p \leq 0.01$) for one or more gene set from the ChIP-seq (red) and TFOE (blue) gene sets, for $\mathsf{cMonkey}_2$ runs that included neither, gene set ("Standard," "NoMotif"), only the ChIP-seq gene set, only the TFOE gene set, and both gene sets, and with motif detection integration included (labeled in bold) or not. Error bars show one standard error over 10 repeated runs.

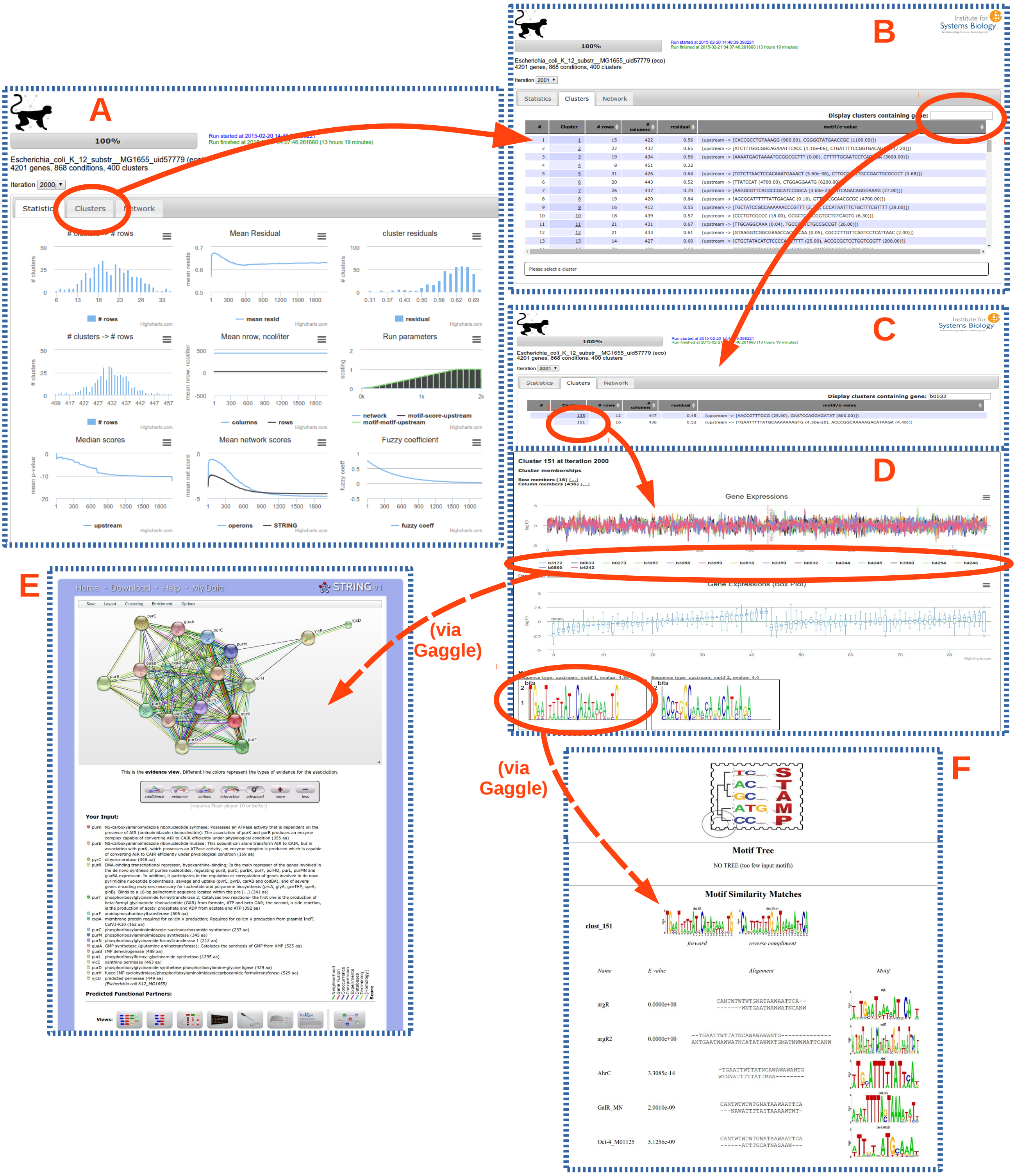

Figure S4. Example cMonkey$_2$ monitoring and visualization web interface and workflow for *E. coli* (screenshots from a web browser). **A.** cMonkey$_2$ run statistics and progress, including histograms of bicluster residuals and sizes, and optimization of scores as a function of iteration. Clicking on the "Clusters" tab shows **B.** a table of all biclusters with individual bicluster statistics, and a gene search field (circled). Upon entering "*b0032*" in the search field, **C.** the two biclusters containing that gene are shown. Clicking on one of the biclusters in the table reveals a visualization of that selected bicluster (**D.**), including gene expression profiles (top), GRE logos (bottom), and GRE positions relative to bicluster gene annotated start sites (not shown). This visualization is populated with FireGoose (2)/ChromeGoose (web browser plug-in) XML microformats, so using this tool enables quick broadcast (indicated with dashed arrows, "via Gaggle") of the bicluster genes to EMBL STRING (**E.**) for gene annotations and/or STAMP (**F.**) which reveals that one of the bicluster predicted GREs is a close match to the binding motif for ArgR (p-value 0.0).

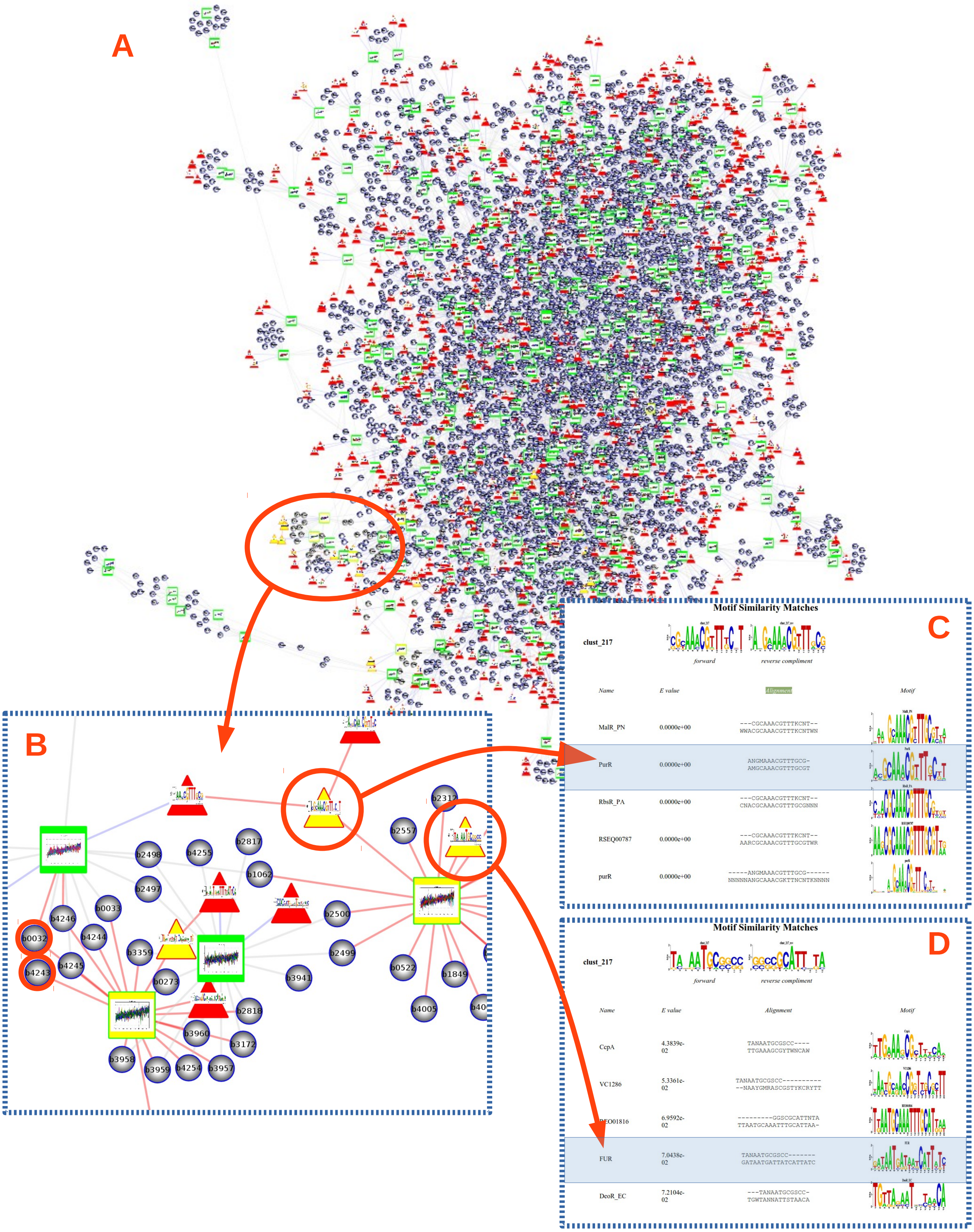

Figure S5. **A.** cMonkey$_2$ *E. coli* bicluster network, rendered in Cytoscape. Genes (circles) and GREs (larger red-framed triangles with motif logos) are connected to biclusters (larger green-framed rectangles with expression profiles) by membership edges. Significantly similar (FDR ≤ 0.01, estimated by Tomtom (3)) GREs are also linked in the network. In this example, the genes *b0032* and *b4246* were highlighted and a subnetwork of neighboring biclusters, genes and GREs extracted (**B.**; selected nodes are highlighted in yellow). Through bicluster membership and broadcast via FireGoose (2)/ChromeGoose to STAMP (see Figure S4), it is revealed (highlighted in light blue) that these genes are part of a regulon that is putatively combinatorially regulated by PurR (p-value ~ 0.0) and possibly Fur (p-value ~ 0.07), in addition to the ArgR GRE described in Figure S4.

REFERENCES

1. Powers, D. M. (2011) Evaluation: from precision, recall and F-measure to ROC, informedness, markedness and correlation. *Journal of Machine Learning Technologies,* **2**(1), 37–63.
2. Bare, J. C., Shannon, P. T., Schmid, A. K., and Baliga, N. S. (2007) The Firegoose: two-way integration of diverse data from different bioinformatics web resources with desktop applications.. *BMC Bioinformatics,* **8**, 456.
3. Gupta, S., Stamatoyannopoulos, J. A., Bailey, T. L., and Noble, W. S. (2007) Quantifying similarity between motifs.. *Genome Biol,* **8**(2), R24.
